# Socioeconomic Inequalities in Uptake of Breast Cancer Screening among Saudi Women: A Cross-Sectional Analysis of a National Survey

**DOI:** 10.3390/ijerph17062056

**Published:** 2020-03-20

**Authors:** Mohammed Khaled Al-Hanawi, Rubayyat Hashmi, Sarh Almubark, Ameerah M. N. Qattan, Mohammad Habibullah Pulok

**Affiliations:** 1 Department of Health Services and Hospital Administration, Faculty of Economics and Administration, King Abdulaziz University, Jeddah 80200, Saudi Arabia; sara-m7@hotmail.com (S.A.); aqattan@kau.edu.sa (A.M.N.Q.); 2 School of Commerce, Centre for Health, Informatics and Economic Research, Faculty of Business, Education, Law and Arts, University of Southern Queensland, Toowoomba, QLD 4350, Australia; rubayyat.hashmi@usq.edu.au; 3 Geriatric Medicine Research (GMR), Nova Scotia Health Authority, Halifax, NS B3H 2E1, Canada; mohammad.pulok@nshealth.ca

**Keywords:** breast cancer, inequality, screening, Saudi Arabia, concentration curve, concentration index

## Abstract

Timely and adequate screening for breast cancer could improve health outcomes and reduce health costs. However, the utilization of free breast cancer screening services among Saudi women is very low. This study aims to investigate socioeconomic inequalities in breast cancer screening among Saudi women. The data of this study were extracted from the nationally representative Saudi Health Interview Survey, conducted in 2013; the study included 2786 Saudi women. Multivariate logistic regression, the concentration curve, and the concentration index were used to examine, illustrate, and quantify income- and education-related inequalities in three outcomes: Knowledge about self-breast examination (SBE), clinical breast examination (CBE) received in the last year, and mammography, that has ever been previously carried out. Results showed a marked socioeconomic gradient in breast cancer screening services. The concentration index by income was 0.229 (SBE), 0.171 (CBE), and 0.163 (mammography). The concentration index by education was 0.292 (SBE), 0.149 (CBE), and 0.138 (mammography). Therefore, knowledge about breast cancer screening, and the utilization of screening services, were more concentrated among richer and better-educated women. Poorer and less educated women had less knowledge about self-breast examination, and had considerably less adherence to clinical breast examination and mammography. The findings are helpful for policy makers to devise and implement strategies to promote equity in breast cancer screening among Saudi women.

## 1. Introduction

Cancer is one of the leading global causes of death [1], and it accounted for an estimated 9.6 million deaths in 2018 [2]. The global prevalence of breast cancer is one of the highest among women [3]. Similarly, in the Kingdom of Saudi Arabia (KSA), breast cancer is the most common type of cancer and the leading cause of cancer deaths among Saudi women [4,5,6]. According to recent Saudi Cancer Registry data, breast cancer ranked the first among women, with 1979 female breast cancer cases [7].

In the past two decades, the breast cancer incidence rate has increased by almost 10-fold in the KSA, and the incidence rate is projected to increase due to population growth and aging [8,9,10]. Although, the burden of disease for breast cancer in the KSA is lower than the average in the Arab region, it is expected to increase given the continuing rise in incidence-rate trends [11,12].

The early detection and appropriate treatment of breast cancer are an important strategy for improving disease prognosis [13]. There are two major components of the early detection of cancer: Education to promote early diagnosis and screening. Efficient utilization of breast cancer screening tests, such as mammography, breast self-examination, and clinical breast examination (CBE) could ensure early diagnoses, and a better chance for treatment and recovery [14]. A mammography test, among others, is recommended for breast cancer screening at least every two years for women aged 50–74 years by the Centers for Disease Control and Prevention [15]. The screening procedure reduces mortality rates and reduces the burden of disease of the healthcare system [12].

Inequalities in breast cancer screening services, due to socioeconomic status (SES), have been found in many settings, with more-deprived women less likely to be screened [16,17,18]. In general, women who are more educated are likely to opt for mammograms, as compared to their less-educated counterparts. Moreover, women in higher occupational classes tend to use screening services more than those in other classes [19]. Socioeconomic inequalities in breast cancer screening are comparatively low in countries with universal access [20].

Government agencies should take relevant measures to deal with these inequalities by organizing screening programs that serve the entire population, regardless of their socioeconomic class. This is necessary for increasing screening attendance and dealing with existing inequalities. However, poverty issues often tend to impede many women from attending to screening sessions [13]. In many developed countries, the highest percentages of women going for screening services are the ones that have a high SES. Moreover, survival rates are higher in this population [21].

Despite free healthcare service provision, including the free provision of breast cancer screening services in the KSA [22], the adoption and utilization of breast cancer screening is still low. In 2013, about 89% of women in the KSA reported not having had a clinical breast examination, and 92% reported never having had a mammogram [5]. Evidence from the KSA showed that personal fears and misconception are the major factors hindering women’s utilization of resources [23]. Other evidence found that education and family history are important predictors of breast cancer screening in the KSA [24,25].

Analysis of the distribution of breast cancer screening by social groups is necessary for formulating policies that promote an equitable uptake of screening. Understanding screening utilization by socioeconomic group helps to devise target-based utilization policies [26,27]. Equitable access to organized screening programs could reduce socioeconomic inequalities in deaths attributed to breast cancer, as found in the city of Florence in Italy [28]. Therefore, analysis of socioeconomic inequalities in breast cancer screening is required to devise policy implications to improve screening utilization, and consequently reducing health-system costs in the KSA. However, no studies from the KSA investigated socioeconomic-related inequalities in the uptake of breast cancer screening. This study contributed to the literature by examining and quantifying income- and education-related inequalities in the awareness about, and utilization of, breast cancer screening services among Saudi women.

## 2. Materials and Methods

### 2.1. Data Source and Sample

This study extracted data from the Saudi Health Interview Survey (SHIS), 2013. The Saudi Ministry of Health collaborated with the Institute for Health Metrics and Evaluation, and the University of Washington to conduct a nationally representative survey. The SHIS collected data on health, healthcare utilization, health outcomes, and sociodemographic characteristics. The survey included questions on sociodemographic characteristics, tobacco use, general health status, physical activities, healthcare access and utilization, different health-related behaviors, and self-reported diagnosed chronic conditions, including hypertension, diabetes, and hypercholesterolemia [29]. A multistage stratified probability sample was employed to recruit survey respondents to ensure the representativity of the Saudi population findings. A total of 10,735 individuals aged over 15 were interviewed out of 12,000 originally contacted households, with a response rate of about 90% in the SHIS. A comprehensive description about the sampling method and data can be found elsewhere [30,31]. This study restricted analysis to women aged 35 years and older, so the main analysis of this study is based on a sample of 2786 women. For mammography screening outcome (see next section), additional analysis was conducted by restricting the sample to women aged 50–74.

### 2.2. Variables

The breast cancer module of the survey is the key research interest of this study. To measure the breast cancer screening utilization, respondents in the SHIS were asked the following questions: “Do you know what a self-breast examination is?”, “How many times during the past 12 months have you self-examined your breasts?”, “How many times during the past 12 months have you visited a doctor’s office or other health professional for a (clinical) breast examination?”, “If you have ever had a mammogram, in what year did you last have a mammogram?”.

The study used these questions to construct three outcome variables of this study: (a) knowledge about self-breast examination, (b) clinical breast exam received in the last year, and (c) ever-done mammography. Knowledge about self-breast examination, which was constructed from first question “Do you know what a self-breast examination is?”, took the value of one if a respondent reported knowledge of what a self-breast examination is, and zero if not. Clinical breast exam received in the last year was constructed from second question “How many times during the past 12 months have you visited a doctor’s office or other health professional for a (clinical) breast examination?”, which was recoded to whether the respondent had received a clinical breast exam. It took the value of one if a respondent had received clinical breast exam, and zero if not. The mammography screening test, which was constructed from third question “If you have ever had a mammogram, in what year did you last have a mammogram?”, was recoded to investigate whether a respondent had ever done a mammogram. It took the value of one if a respondent had ever done a mammogram, and zero if not.

This study used the education of women and monthly household income as SES proxies. Education was categorized as primary education or less (reference category), secondary education, and tertiary education. Primary education is the first stage of formal education, coming after pre-school and before secondary school. Secondary education includes intermediate school and high school. Tertiary education is post-secondary education, it is the educational level following the completion of a school providing a secondary education.

Household monthly income (in Saudi riyal (SR)) was grouped into six categories: less than SR 3000 (reference category); SR 3000 to less than SR 5000; SR 5000 to less than SR 7000; SR 7000 to less than SR 10,000; SR 10,000 to less than SR 15,000; and SR 15,000 or more. Other variables included in the analysis were age, labor-force status, and marital status. The age variable was entered in categories: 35–49 (reference category), 50–59, 60–69, 70–74, and 75 or above. The labor-force status was split into categories of employed (reference category), unemployed, and out of the labor force. Marital status was also split into categories: Married (reference category), unmarried, and divorced.

### 2.3. Statistical Analysis

Analysis started by descriptive analysis on outcome variables and sociodemographic variables, and a cross tabulation of the outcome variables by SES variables. Multivariate logistic regression models were estimated to examine the association between outcome variables and independent variables. The concentration curve (CC) and concentration index (C) were used to visualize and quantify inequality breast cancer screening by SES [32,33]. To construct the CC, the cumulative share of outcome variables was plotted against the cumulative percentage of women ranked from lowest to highest. If the outcome were more concentrated among poorer (less-educated) women, the CC lay above the 45-degree line or line of equality. In this case, inequality was pro-poor (in favor of the less-educated). However, if the outcome were concentrated more among richer (educated) women, then the CC was below the diagonal. If the CC exactly equaled the 45-degree line, there was perfect equality in the outcome variable with respect to the SES.

The CC depicts but does not measure SES-related inequality. Therefore, C was estimated, which equaled twice the area between CC and line of equality. The value of C fell between −1 and 1. A positive (negative) C indicated that the distribution of outcome variables was concentrated among people with high (low) SES. When the C was zero, it implied no socioeconomic-related inequality. However, in the case of binary outcome measures, upper and lower C bounds may not lie between −1 and 1 [34]. The bounds were influenced by the mean of the outcome variable. Hence, the study used Wagstaff and Erreygers’ approaches to account for this issue [35,36]. Stata/MP version 15.1(StataCorp LLC. College Station, TX, USA) was used to conduct all statistical analyses in this study.

### 2.4. Ethical Clearance

The survey protocol was approved by the Saudi Ministry of Health and its Institutional Review Board (IRB). The participants consented and agreed to participate in the study. They were informed that participation is voluntary, and they could withdraw at any time. Furthermore, they were informed that the data would be used for the purpose of research at a future time point. After all the data were collected, they were fully anonymized by the Ministry of Health before we accessed them. Thus, it is not possible to identify people using the current data.

## 3. Results

Table 1 shows that about 70% of the respondents reported not having knowledge about self-examination of breast cancer. About 11% of the women reported that they had had a clinical breast examination in the previous year, and only 6.5% of women reported that they had ever had a mammogram. Table 2 reports the demographic and socioeconomic characteristics of the study population. About 60% of the women were aged between 35–49 years, and more than 70% of the women were married. One quarter of the women belonged to households in the lowest income category, that is, less than SR 3000. More than half of the women had primary education or less. About 56% of women were out of the labor-force, one quarter were employed, and approximately 20% were unemployed.

**Figure 1:**
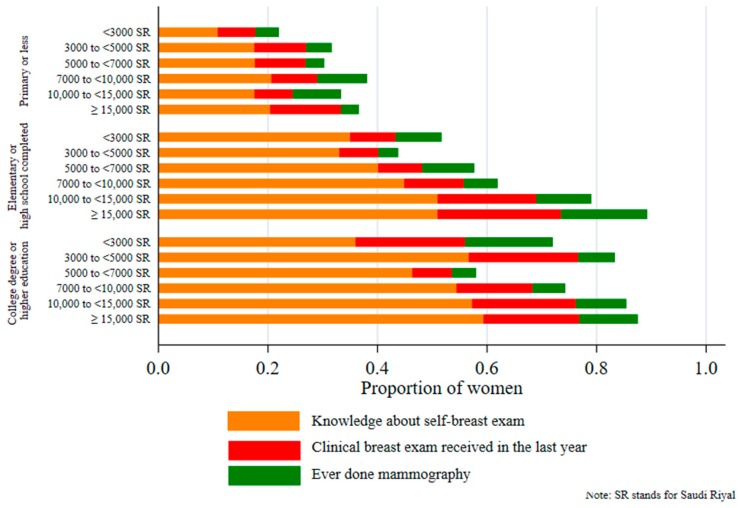
Distribution of breast cancer screening uptake by education and income.

**Figure 2:**
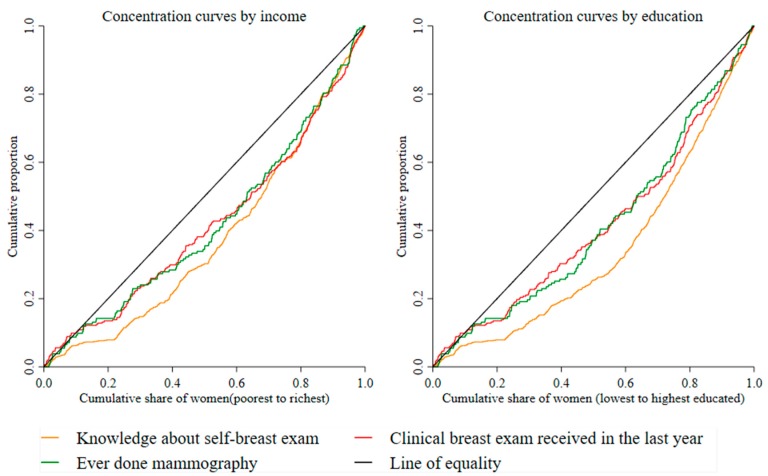
Concentration curves of breast cancer screening uptake by education and income.

Figure 1 reveals a marked socioeconomic gradient in the uptake of breast cancer screening. The proportion of breast cancer screening uptake was significantly lower in women from poorer households and women with less education. For example, about 60% of women from households with a monthly income of SR 15,000 or more, with a college-degree and higher education, reported to have knowledge about self-breast examination, compared with only 10% women with primary education in households with a monthly income of SR 3000 or less. Figure 2 plots the CCs of the three outcomes by income and education. All CCs were below the line of equality, indicating pro-rich and pro-educated inequalities in the uptake of breast cancer screening among Saudi women. CCs by education were farther away from the line of equality, showing a greater extent of education-related inequalities.

Table 3 and Table 4 present the estimates of socioeconomic inequalities measured by the standard concentration index (C), Wagstaff’s index (W), and Erreygers’s index (E). All estimates were positive and statistically significant (*p* < 0.001). This suggests that inequalities in the three outcomes were in favor of women from richer households and better-educated women. These findings confirm inequalities illustrated by the CCs. Overall, the magnitudes of education-related inequality were higher than income-related inequalities in breast cancer screening uptake. Inequalities in the self-examination of breast cancer screening were also higher than inequalities in the two other outcomes. Results remained similar when the Erreygers and Wagstaff methods were applied to measure inequalities. Table A1 in the Appendix A reports the inequality estimates for the mammography variable for the sample of women aged 50–74, which revealed similar results.

Unadjusted odds ratio (OR) and adjusted odds ratio (AOR) obtained from the logistic regression models that examine the correlates of the uptake of breast cancer screening are shown in Table 5 (results for mammography are reported in Table A2 for the sample of women aged 50–74). Higher-income groups had a gradually increasing odds ratio with regard to knowledge about self-breast examination. However, only the highest-income group (≥SR 15,000) was significant at the 1% level for clinical breast examination received in the last year when the odds ratios were adjusted (AOR: 1.749, 95% CI: 1.108–2.762). For ever-done mammography, odds ratios were significant for women belonging to households of an income category of SR 10,000 to less than SR 15,000 (OR: 1.927, 95% CI: 1.193–3.114), and for women belonging to households of an income category of ≥SR 15,000 (OR: 2.128, 95% CI: 1.311–3.453). However, in the adjusted model, there was no significant association between ever-done mammography and income except the highest-income group (≥SR 15,000) which was significant at the 10% level.

AORs increased as the level of education increased for the outcome of having knowledge about self-breast examination (secondary education AOR: 2.794, 95% CI: 2.199–3.55; tertiary education AOR: 3.683, 95% CI: 2.731–4.968). For ever-done mammography, AORs were significant and similar for secondary (AOR: 1.997, 95% CI: 1.302–3.064) and tertiary (AOR: 1.907, 95% CI: 1.117–3.255) education. The likelihood of clinical breast examination was higher among better-educated women (secondary education OR: 1.542, 95% CI: 1.149–2.069; tertiary education OR: 2.142, 95% CI: 1.609–2.85). They were not significant, however, if the odds ratios were adjusted. The adjusted odds ratios were lower than those for knowledge on self-breast examination (AOR: 0.782, 95% CI: 0.612–0.998) and clinical breast examination (AOR: 0.686, 95% CI: 0.487–0.968) in the out-of-labor-force category. Analysis for the restricted sample revealed no changes in the results for the ever-done mammography outcome.

## 4. Discussion

To the best of our knowledge, the present study is the first to examine and quantify, using nationally representative data, socioeconomic inequalities in the uptake of breast cancer screening among Saudi women. Despite the free provision of healthcare services to the Saudi population in the KSA [22], our results suggested socioeconomic inequalities favored better-off women in the uptake of breast cancer screening practices. Poorer and less-educated women had less knowledge about self-breast examination, and had considerably less adherence to clinical breast examination and mammography. These findings were also confirmed by estimates of income- and education-related inequalities in breast cancer screening uptake. Findings from logistic regression analysis further elucidated that income is not a significant predictor of adherence to mammography screening, but significant in higher-income groups for knowledge about self-breast examination and clinical breast examination. We found education to be a significant predictor of the three measurements of breast cancer screening uptake.

Our findings showed that education was the most important determinant of breast cancer screening uptake. Since women in the KSA are very conservative, they might be more likely to shy away from clinical breast examination and mammography [5]. Findings indicated that knowledge of self-breast examination was more concentrated among educated women. Although, evidence on the effectiveness of self-breast examination in detecting breast cancer screening is contestable [14], it could be considered to be one of the tools in a conservative society as in the KSA. Educated women would understand the importance of breast cancer screening, and would be more willing to adhere to breast cancer screening. A possible solution for the KSA government in reducing screening inequality and increase uptake would be in improving health promotion based on the cultural context of the KSA. Moreover, establishing educational breast cancer campaigns is warranted. Evidence has shown that promoting awareness about breast cancer could increase the uptake of mammography and breast self-examination behaviors, and increase the likelihood of breast cancer screening attendance [37].

Compliance with mammography practices among Saudi women is low compared to European or other Western countries [21,38]. This is because the Arab regional context is different, and regional sociodemographic, cultural, religious and traditional belief systems have to be accounted for, in order to improve breast cancer screening practices [39,40]. This study revealed quantitative measurements in this regard, and recommends health promotion in poorer and less-educated women to increase screening efficacy.

The study results could be used as a comparison for other Arab and Middle Eastern countries, especially in the Arabian Gulf region, which share a similar cultural and socioeconomic context for their screening-efficacy efforts. Generally, findings related to the uptake of breast cancer screening have been inconsistent around the world. It is, therefore, recommended that a systematic review of current studies are carried out, in order to identify the limitations, and offer the right basis for future research policy and directions [41].

Despite free healthcare services in the KSA, results showed the low utilization rate of breast cancer screening services. This can be explained by the lack of knowledge, awareness, and information about breast cancer and its prevention. Several studies revealed the low awareness of breast cancer among Saudi women [42,43,44]. Another study on the knowledge and practices of breast cancer screening among Saudi women reported a very low utilization rate of breast cancer screening uptake, and concluded that breast cancer screening is free in Saudi Arabia, but there are still almost no takers [5]. Hence, it is recommended that educational campaigns about breast cancer and its prevention stress the benefits of mammography screening tests and free access, and they must be drafted and implemented to increase the relevance of screening among Saudi women. This step is a public-health imperative.

Few limitations should be considered to interpret the findings of this study. Data used in analysis in this study were self-reported, which might suffer from reporting bias. Future studies could employ administrative data to address this issue [45]. We also acknowledge the limitation to use ever-done mammography as a screening outcome. It is recommended that mammography should be performed biennially to effectively detect symptoms of breast cancer. However, there is no information available regarding the performance of routine mammography in the SHIS 2013. Since this was a cross-sectional study, it precludes interpretation of the causal relationship between screening uptake and the SES of women. The measure of SES (income and education) used in this study was collected as grouped data from the SIHS. This might have caused a downward bias to quantify SES-related inequalities, as variation within the SES groups was masked in the analysis [46]. Therefore, our results might suggest an underestimation of SES-related inequality.

## 5. Conclusions

Rising prevalence of breast cancer among Saudi women is a major public-health concern. Early detection and appropriate treatment of breast cancer would help to minimize the mortality rate among Saudi women. However, designing appropriate breast cancer screening intervention is very complex in nature [14]. A recent viewpoint of the International Agency for Research on Cancer (IARC) working group re-evaluated breast cancer screening practices concerning over diagnosis of breast cancer [47]. In addition, improvement in treatment outcomes for late-stage breast cancer calls for careful consideration of screening practices [48]. Given this background, the objective of this paper was to offer an equity perspective of breast cancer screening in the KSA. We found a low utilization of breast cancer screening services, and a marked socioeconomic gradient in knowledge about self-breast examination and the uptake of screening services among the Saudi women. We found income- and education-related inequalities in breast cancer screening favoring better-off Saudi women. Given the free healthcare service provision in the KSA, these inequalities are unfair and require policy considerations. The findings of this study offer important implications to design policies for the equitable use of breast cancer screening in the KSA. For example, efforts should be made toward establishing an organized population-based screening program to ensure equity and effectiveness [49]. The supply-side factor should ascertain age-appropriate and need-based provisions of high-quality screening services. To warrant equitable service utilization from the demand side, educational campaigns and community-based programs could be undertaken to reduce socioeconomic inequities in the use of breast cancer screening services. The government could also introduce awareness programs that might enhance the early detection of breast cancer and the benefits of screenings. However, this paper did not identify the causes of socioeconomic inequality; future studies could investigate this unexplored area. Further research, is thus, required to develop equitable screening interventions.

## Figures and Tables

**Table 1:** Knowledge and uptake of breast cancer screening among Saudi women 35+ years old.

Outcome Variables	%	Frequency	95% CI
**Knowledge about self–breast exam**			
No	69.99	1950	(68.26–71.67)
Yes	30.01	836	(28.33–31.74)
**Clinical breast exam received in the last year**			
No	89.09	2482	(87.87–90.19)
Yes	10.91	304	(9.81–12.13)
**Ever-done mammography**			
No	93.43	2603	(92.45–94.29)
Yes	6.57	183	(5.71–7.55)

**Table 2:** Demographic and socioeconomic characteristics of Saudi women 35+ years old.

Variables	%	Frequency	95% CI
**Age**			
35–49 years	59.26	1651	(57.42–61.07)
50–59 years	21.18	590	(19.7–22.74)
60–69 years	11.2	312	(10.08–12.43)
70–74 years	3.55	99	(2.93–4.31)
75+ years	4.81	134	(4.07–5.67)
**Income**			
<SR 3000	24.95	695	(23.37–26.59)
SR 3000 to less than SR 5000	17.59	490	(16.22–19.05)
SR 5000 to less than SR 7000	15.94	444	(14.62–17.34)
SR 7000 to less than SR 10,000	14.47	403	(13.21–15.82)
SR 10,000 to less than SR 15,000	14.32	399	(13.07–15.67)
≥SR 15,000 SR	12.74	355	(11.55–14.03)
**Education**			
Primary or less	55.92	1558	(54.07–57.76)
Secondary	23.62	658	(22.08–25.23)
Tertiary	20.46	570	(19–22)
**Labor-force status**			
Employed	25.31	705	(23.72–26.95)
Unemployed	18.49	515	(17.09–19.97)
Out of labor force	56.21	1566	(54.36–58.04)
**Marital status**			
Married	71.86	2002	(70.16–73.5)
Unmarried	2.73	76	(2.18–3.4)
Divorced *	25.41	708	(23.83–27.06)

Note: Divorced group includes separated and widowed individuals.

**Table 3:** Inequality indices in uptake of breast cancer screening by income.

Title	C	95% CI	W	95% CI	E	95% CI
Knowledge about self-breast examination	0.229 ***	(0.199–0.260)	0.327 ***	(0.284–0.371)	0.275 ***	(0.238–0.312)
Clinical breast exam received in last year	0.171 ***	(0.109–0.234)	0.192 ***	(0.122–0.263)	0.075 ***	(0.047–0.102)
Ever-done mammography	0.163 ***	(0.079–0.246)	0.174 ***	(0.085–0.264)	0.043 ***	(0.021–0.065)

Note: 95% confidence intervals in parentheses. Significance level * *p* < 0.10, ** *p* < 0.05, and *** *p* < 0.01. C = standard concentration index, W = Wagstaff’s index, E = Erreygers’s index.

**Table 4:** Inequality indices in uptake of breast cancer screening by education.

Title	C	95% CI	W	95% CI	E	95% CI
Knowledge about self-breast exam	0.292 ***	(0.263–0.320)	0.417 ***	(0.376–0.458)	0.350 ***	(0.316–0.385)
Clinical breast exam received in the last year	0.149 ***	(0.091–0.208)	0.168 ***	(0.102–0.233)	0.065 ***	(0.040–0.091)
Ever-done mammography	0.138 ***	(0.063–0.213)	0.148 ***	(0.067–0.228)	0.036 ***	(0.017–0.056)

Note: 95% confidence intervals in parentheses. Significance level * *p* < 0.10, ** *p* < 0.05 and *** *p* < 0.01. C = standard concentration index, W = Wagstaff’s index, E = Erreygers’s index.

**Table 5:** Logistic regression results of the correlates of breast cancer screening uptake.

Variables	Knowledge about Self-Breast Examination				Clinical Breast Exam Received in the Last Year				Ever-done Mammography			
	OR	95% CI	AOR	95% CI	OR	95% CI	AOR	95% CI	OR	95% CI	AOR	95% CI
**Age**												
35–49 years (ref)	1		1		1		1		1		1	
50–59 years	0.526	(0.426–0.65) ***	0.865	(0.679–1.102)	0.919	(0.686–1.23)	1.081	(0.782–1.496)	1.053	(0.729–1.521)	1.256	(0.835–1.89)
60–69 years	0.192	(0.133–0.278) ***	0.412	(0.274–0.619) ***	0.483	(0.3–0.778) ***	0.623	(0.367–1.057) *	0.992	(0.612–1.607)	1.276	(0.722–2.257)
70–74 years	0.049	(0.015–0.156) ***	0.104	(0.032–0.339) ***	0.375	(0.151–0.933) **	0.452	(0.172–1.185)	0.283	(0.069–1.164) *	0.309	(0.071–1.342)
75+ years	0.113	(0.057–0.224) ***	0.238	(0.115–0.492) ***	0.331	(0.144–0.76) ***	0.401	(0.164–0.977) **	0.644	(0.278–1.493)	0.703	(0.277–1.785)
**Income**												
<SR 3000 (ref)	1		1		1		1		1		1	
SR 3000 to less than SR 5000	1.913	(1.417–2.583) ***	1.252	(0.905–1.732)	1.312	(0.868–1.982)	1.081	(0.701–1.666)	0.886	(0.513–1.531)	0.849	(0.48–1.502)
SR 5000 to less than SR 7000	2.555	(1.898–3.44) ***	1.314	(0.945–1.827)	1.157	(0.748–1.79)	0.903	(0.567–1.438)	1.078	(0.632–1.837)	0.934	(0.527–1.653)
SR 7000 to less than SR 10,000	3.819	(2.844–5.128) ***	1.633	(1.168–2.282) ***	1.477	(0.966–2.257) *	1.065	(0.668–1.699)	1.462	(0.88–2.431)	1.201	(0.683–2.11)
SR 10,000 to less than SR 15,000	4.975	(3.714–6.664) ***	1.769	(1.253–2.497) ***	2.232	(1.507–3.305) ***	1.488	(0.943–2.347) *	1.927	(1.193–3.114) ***	1.588	(0.909–2.773)
≥ SR 15,000	5.48	(4.061–7.396) ***	1.879	(1.324–2.665) ***	2.668	(1.802–3.95) ***	1.749	(1.108–2.762) ***	2.128	(1.311–3.453) ***	1.701	(0.972–2.977) *
**Education**												
Primary or less (ref)	1		1		1		1		1		1	
Secondary	4.119	(3.349–5.065) ***	2.794	(2.199–3.55) ***	1.542	(1.149–2.069) ***	1.215	(0.861–1.715)	1.824	(1.279–2.602) ***	1.997	(1.302–3.064) ***
Tertiary	6.736	(5.434–8.35) ***	3.683	(2.731–4.968) ***	2.142	(1.609–2.85) ***	1.234	(0.81–1.879)	1.809	(1.247–2.624) ***	1.907	(1.117–3.255) **
**Labor-force status**												
Employed (ref)	1		1		1		1		1		1	
Unemployed	0.35	(0.273–0.447) ***	1.302	(0.955–1.776) *	0.534	(0.374–0.762) ***	0.841	(0.549–1.289)	1.144	(0.751–1.743)	1.99	(1.19–3.327) **
Out of labor force	0.296	(0.245–0.358) ***	0.782	(0.612–.998) **	0.495	(0.38–0.644) ***	0.686	(0.487–0.968) **	0.748	(0.524–1.066)	1.167	(0.744–1.833)
**Marital status**												
Married (ref)	1		1		1		1		1		1	
Unmarried	0.712	(0.424–1.196)	0.633	(0.365–1.097)	0.43	(0.156–1.188)	0.433	(0.155–1.208)	0.38	(0.092–1.564)	0.399	(0.096–1.659)
Divorced	0.522	(0.426–0.64) ***	1.174	(0.915–1.507)	0.863	(0.651–1.143)	1.242	(0.901–1.712)	1.022	(0.726–1.438)	1.363	(0.916–2.026)

Note: 95% confidence intervals in parentheses. Significance level * *p* < 0.10, ** *p* < 0.05, and *** *p* < 0.01. OR = odds ratio; AOR = adjusted odds ratio.

## Appendix A

**Table A1:** Inequality indices in mammography screening (age 50–74, *n* = 1001).

SES Indicators	C	95% CI	W	95% CI	E	95% CI
Income-related	0.169 **	(0.031–0.307)	0.180 **	(0.033–0.328)	0.044 **	(0.008–0.080)
Education-related	0.201 ***	(0.083–0.320)	0.215 ***	(0.089–0.342)	0.052 ***	(0.022–0.083)

Note: 95% confidence intervals in parentheses. Significance level * *p* < 0.10, ** *p* < 0.05, and *** *p* < 0.01. C = standard concentration index, W = Wagstaff’s index, E = Erreygers’s index.

**Table A2:** Logistic regression results of the correlates of mammography screening (age 50–74, *n* = 1001).

Variables	Ever-Done Mammography			
	OR	95% CI	AOR	95% CI
**Age**				
50–59 years	1		1	
60–69 years	0.942	(0.547–1.620)	1.096	(0.597–2.010)
70–74 years	0.269	(0.064–1.130) *	0.265	(0.059–1.184) *
**Income**				
<SR 3000 (ref)	1		1	
SR 3000 to less than SR 5000	0.980	(0.428–2.242)	0.956	(0.403–2.267)
SR 5000 to less than SR 7000	0.991	(0.405–2.464)	0.750	(0.292–1.927)
SR 7000 to less than SR 10,000	2.210	(1.023–4.775) **	1.544	(0.648–3.679)
SR 10,000 to less than SR 15,000	3.065	(1.456–6.455) ***	1.913	(0.797–4.592)
≥SR 15,000	1.391	(0.532–3.635)	0.868	(0.306–2.462)
**Education**				
Primary or less (ref)	1		1	
Secondary	3.607	(1.942–6.700) ***	3.695	(1.789–7.633) ***
Tertiary	2.820	(1.261–6.310) **	3.027	(1.120–8.179) **
**Labor-force status**				
Employed (ref)	1		1	
Unemployed	1.453	(0.570–3.707)	3.181	(1.062–9.524) **
Out of labor force	0.838	(0.344–2.043)	1.498	(0.545–4.119)
**Marital status**				
Married (ref)	1		1	
Unmarried	0.794	(0.103–6.115)	0.621	(0.075–5.173)
Divorced	0.992	(0.594–1.658)	1.394	(0.777–2.502)

Note: 95% confidence intervals in parentheses; significance level * *p* < 0.10, ** *p* < 0.05, and *** *p* < 0.01. OR = odds ratio; AOR = adjusted odds ratio.
